# Use of a sealant to prevent prolonged air leaks after lung resection: a prospective randomized study

**DOI:** 10.1186/1749-8090-7-106

**Published:** 2012-10-08

**Authors:** Cosimo Lequaglie, Gabriella Giudice, Rita Marasco, Aniello Della Morte, Massimiliano Gallo

**Affiliations:** 1 Department of Thoracic Surgery, IRCCS-CROB Centro Riferimento Oncologico Basilicata, Rionero in Vulture, PZ, Italy; 2 Basilicata Regional Health Department, Potenza, Italy; 3 Department of Thoracic Surgery, IRCCS-CROB Centro Riferimento Oncologico Basilicata, Via Padre Pio n° 1, 85028, Rionero in Vulture, PZ, Italy

**Keywords:** Air leak, Surgical sealant, Digital chest drain

## Abstract

**Background:**

Pulmonary air leaks are common complications of lung resection and result in prolonged hospital stays and increased costs. The purpose of this study was to investigate whether, compared with standard care, the use of a synthetic polyethylene glycol matrix (CoSeal®) could reduce air leaks detected by means of a digital chest drain system (DigiVent™), in patients undergoing lung resection (sutures and/or staples alone).

**Methods:**

Patients who intraoperatively showed moderate or severe air leaks (evaluated by water submersion tests) were intraoperatively randomized to receive just sutures/staples (control group) or sutures/staples *plus* CoSeal® (sealant group). Differences among the groups in terms of air leaks, prolonged air leaks, time to chest tube removal, length of hospital stay and related costs were assessed.

**Results:**

In total, 216 lung resection patients completed the study. Nineteen patients (18.1%) in the control group and 12 (10.8%) patients in the sealant group experienced postoperative air leaks, while a prolonged air leak was recorded in 11.4% (n = 12) of patients in the control group and 2.7% (n = 3) of patients in the sealant group. The difference in the incidence of air leaks and prolonged air leaks between the two groups was statistically significant (p = 0.0002 and p = 0.0013). The mean length of hospital stay was significantly shorter in the sealant group (4 days) than the control group (8 days) (p = 0.0001). We also observed lower costs in the sealant group than the control group.

**Conclusion:**

The use of CoSeal® may decrease the occurrence and severity of postoperative air leaks after lung resection and is associated with shorter hospital stay.

**Trial registration:**

Not registered**.** The trial was approved by the Institutional Review Board of the IRCCS-CROB Basilicata Regional Cancer Institute, Rionero in Vulture, Italy.

## Background

Pulmonary air leaks are a common complication in patients undergoing elective lung resection and result in prolonged hospital stays and greater healthcare costs [1]. ‘Prolonged’ air leaks (PAL) have previously been defined as leakages lasting beyond the 7^th^ postoperative day [2]. However, with the aim of safely discharging patients on the 4^th^–5^th^ postoperative day, it has been suggested that the definition of PAL should be modified to include any leak lasting longer than five days [3].

Major risk factors for developing pulmonary air leaks include emphysema, diabetes mellitus, incomplete interlobar fissures, the presence of pleural adhesions, and low levels of serum albumin and cholinesterase; other factors that can cause pulmonary air leaks include the creation of new fissures, dissection of adhesions, manipulation of the lung, and suturing with staplers [4].

Despite the use of techniques developed to minimize the occurrence of air leaks, such as pleural tenting after upper lobe resection, phrenic nerve crush, pneumoperitoneum and fissureless surgery [5-8], PALs still have an incidence ranging from 15 to 18% after routine pulmonary resection and are often accompanied by a series of events, that may have a negative impact on the patient’s recovery, prolong the length of stay and raise hospital costs. Complications such as these underlie the high level of interest shown in the international literature for a phenomenon that could be considered as widespread as it is ambiguous.

The preferred method to reduce air leaks is to prevent them from occurring; therefore, care should be taken to perform an accurate dissection of the structures along well-defined anatomical planes between the segments and lobes of the lung. Nevertheless, attention has focused on modifying chest drains to allow accurate measurement of transpleural air flows and towards the use of products that aim to prevent parenchymal air leaks.

The use of traditional chest drains to detect air leaks is essentially analogical, as it is based on the principle of identifying air bubbles on the surface of a water valve and thus is largely dependent on the observers’ impressions. Recently, digital pleural drainage units, the prototype of which is the DigiVent™ (Millicore A.B., Sweden), have allowed measurement and direct estimation of transpleural air flows (in ml/min) and pleural pressures (p_i_, inspiratory pressure and p_e_, expiratory pressure, in cm H_2_O), measured extemporaneously and according to a cumulative reading taken over 1, 3 and 6 h. Further details of the characteristics of the DigiVent™ system have been described previously [9-13].

The treatment of air leaks ranges from direct suture with thread or staples, to cauterization or laser vaporization; or from the simple use of Heimlich valves until the leak resolves, to the use of biological or synthetic glues and sealants. A number of different types of surgical sealant have been developed to prevent or reduce postoperative alveolar air leaks, including: fibrin sealants, collagen fleeces, and glutaraldehyde- or polyethylene glycol (PEG)-based synthetic glues. Surgical sealants are particularly useful in cases where PAL cannot be controlled by sutures or staples alone.

Of the available surgical sealants, much of the literature has focused on CoSeal® (Baxter Healthcare, Deerfield, IL, USA), a biocompatible PEG polymer. CoSeal® is composed of two synthetic PEGs: a dilute hydrogen chloride solution and a sodium phosphate/carbonate solution. At the time of administration, the solutions combine to form a hydrogel, which cross-links with proteins, causing immediate adherence to the tissues. The sealant is completely absorbed by the body within 30 days of application [14].

The aim of this prospective, randomized study was to evaluate the role of a synthetic PEG matrix, CoSeal®, in reducing parenchymal air leaks detected by a digital chest drainage system, DigiVent™, compared with standard treatment (suture and/or staples).

## Methods

### Patients

The study was conducted between March 2008 and December 2011. A total of 1080 consecutive patients who had undergone lung resections were enrolled. The trial was approved by the Institutional Review Board of the IRCCS-CROB Basilicata Regional Cancer Institute, Rionero in Vulture, Italy, and was performed in accordance with best practice in Italy. All patients provided written informed consent prior to enrollment.

The inclusion criteria were: males or females ≥18 years of age undergoing lung resection (bilobectomy or lobectomy, anatomical and atypical segment resection) or pleurectomy/decortication. Exclusion criteria were: immunodeficiency, patients undergoing bronchoplastic procedures, and known hypersensitivity to any component of the investigational products. Although CoSeal is contraindicated in pleural decortications, we did not exclude these patients (n = 3) because in our long experience we have not experienced any problems with increased air leaks or inflammatory complications.

### Surgical procedures

Resectability was evaluated by computed tomography (CT) scan, bronchoscopy and mediastinoscopy, if indicated. Operability was assessed by arterial blood gas analysis, pulmonary function tests, electrocardiogram and echocardiography.

All pulmonary resections were performed at a single institution by one of four attending thoracic surgeons through an anterior-lateral muscle-sparing thoracotomy in the 4^th^–5^th^ intercostal space, or a video-assisted thoracoscopy. Mechanical staplers (EndoGIA 30 or 45–4.8 mm) were used to develop incomplete fissures and close the bronchus (transverse anastomosis [TA] 30 mm). A complete hilar-mediastinal lymphadenectomy was performed in all patients.

After re-inflation of the operated lung (peak pressure of 25 cm H_2_O), air leaks were detected by immersing the lung in saline and rated as: 0 = no evidence of air leak; 1 = moderate air leaks, characterized by non-coalescent single bubbles; and 2 = severe air leaks, with coalescent bubbles.

Patients with moderate or severe air leaks were randomized to one of two groups: control group, in which intraoperative air leaks were treated by direct manual or stapler suture; or sealant group, in which air leaks were treated with sutures/staples plus CoSeal®. Following the submersion test for air leaks, the surface to be treated was dried to allow proper adherence of the sealant. The sealant was administered on the suture lines or lung surfaces identified as the source of leakage and a second test for air tightness was performed; if there was still an air leak, the sealant was reapplied and a third test was subsequently conducted. In patients in the control group, the persistence or absence of an air leak was registered without further intervention. This study design was chosen to best reflect routine surgical practice, where lung surgery patients who receive standard care (sutures/staples and no sealant application) are closed up without further intervention, even if an intraoperative air leak persists. Similarly, in the sealant group, the possibility for a second application of the product reflects the standard practice of sealant use by surgeons.

### Chest tube management

Two 28 French multi-fenestrated chest tubes were positioned before the closure of the thoracotomy after lobectomies, bilobectomies, and decortications: one anteriorly near the apex and one in a postero-basal position. Patients who underwent wedge resection had only one drain inserted, with the exception of cases of severe bullous emphysema.

Chest drains were examined twice daily (once in the morning and once in the afternoon) and withdrawn if the volume of drained fluid was ≤250 ml in 24 h; provided there was no frank blood, the mean transpleural air flow recorded by the digital system was <20 ml/min, instantaneous air flow spikes were ≤200 ml/min and radiological features excluded a pneumothorax >20% of the operated hemithorax.

### Randomization

Patients in which a moderate or severe air leak was observed before thoracotomy closure were randomized (1:1). Randomization was performed by a shuffled, sealed envelope technique and envelopes containing the group allocation were opened in the operating room.

### Statistical analysis

The following postoperative variables were considered: incidence of postoperative air leaks, defined as mean air flow ≥20 ml/min recorded by the DigiVent™ software, evidence of prolonged air leaks (defined as an air leak lasting ≥5 days) and length of hospital stay.

Results are given as mean values. For categorical variables, the statistical significance of differences between the control group and sealant group were determined using the Chi-square test. Numerical variables were compared using an unpaired Students *t* test. A p-value of <0.05 was considered significant.

## Results

### Study population

**Figure 1:**
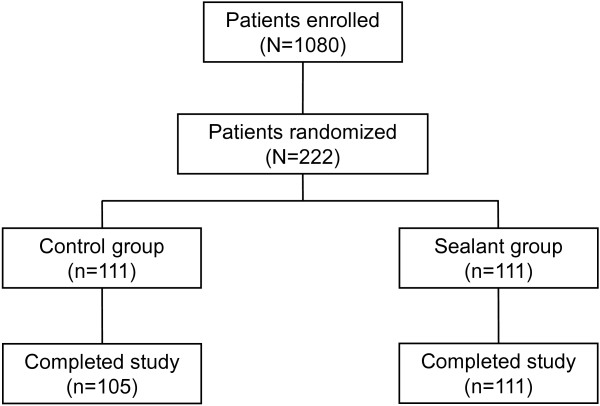
Patient flow through the study.

Of the 1080 patients enrolled in the study, 222 patients were randomized (1:1) to control (n = 111) or sealant (n = 111) (Figure 1). A total of six patients in the control group were excluded from the analysis due to incomplete postoperative data. Therefore, a total of 105 patients were included in the analysis for the control group (n = 71 males and n = 34 females) and a total of 111 patients (n = 65 males and n = 46 females) for the sealant group.

The two treatment groups were comparable in terms of gender distribution, mean age, ‘pack years’ (number of years as a smoker, multiplied by the average number of cigarettes smoked per day), mean preoperative forced expiratory volume in one second (FEV_1_), mean predicted postoperative FEV_1_ (ppoFEV_1_), pathology and surgical procedures, as shown in Tables 1, 2 and 3.

**Table 1:** Patient characteristics

**Variable**	**Control group (N = 111)**	**Sealant group (N = 111)**	**p*-*value**
Age (years)	67.5	65.3	0.1
FEV_1_ (%)	78.3	82.7	0.4
ppoFEV_1_ (%)	62.5	65.4	0.2
paO_2_ (mmHg)	82.1	80.3	0.3
paCO_2_ (mmHg)	39.2	38.6	0.5
Pack years*	48.0	46.7	0.05
Side (% right)	57.4	56.6	0.7^a^
Site (% upper lobes)	78.1	73.2	0.05^a^
Pleural adhesions (%)	43.2	40.5	0.1^a^
Stapler suture length (cm)	62.9	64.8	0.4

*The number of pack-years was calculated as the total number of years as a smoker multiplied by the average number of cigarettes smoked per day, divided by 20.

^a^Chi-square test.

Results are mean values and based on the total population, unless stated otherwise.

FEV_**1**_ and ppoFEV_**1**_ are expressed as percentage of predicted values for age, gender and height.

*FEV*_***1***_ forced expiratory volume in one second, *ppoFEV*_***1***_ predicted postoperative FEV_**1**_, *paO*_*2*_ partial pressure of oxygen, *paCO*_*2*_ partial pressure of carbon dioxide.

**Table 2:** Patient characteristics: lung diseases

**Diagnosis**	**Control group n (%)**	**Sealant group n (%)**
**Total n, (%)**	105 (100.0)	111 (100.0)
**Malignant disease**	75 (71.5)	82 (73.9)
Primary lung neoplasm	52 (49.5)	47 (42.3)
Adenocarcinoma	33 (31.4)	18 (16.2)
Epidermoid	18 (17.1)	16 (14.4)
Bronchoalveolar	10 (9.52)	5 (4.50)
Poorly differentiated	5 (4.76)	3 (2.70)
Adenosquamous	3 (2.86)	1 (0.90)
Neuroendocrine	3 (2.86)	1 (0.90)
Typical carcinoid	3 (2.86)	2 (1.80)
Atypical carcinoid	0 (0.0)	1 (0.90)
Metastasis	23 (21.9)	35 (31.5)
Kidney	2 (1.90)	4 (3.60)
Ovarian	1 (0.95)	7 (6.31)
Skin	1 (0.95)	3 (2.70)
Salivary ducts	2 (1.90)	1 (0.90)
Prostatic	2 (1.90)	3 (2.70)
Breast	14 (13.3)	11 (9.91)
Colorectal	2 (1.90)	6 (5.40)
**Benign disease**	30 (28.6)	29 (26.1)
Flogistic	23 (21.9)	23 (20.7)
Disonthogenetic	5 (4.76)	5 (4.50)
Hydatid cyst	2 (1.90)	1 (0.90)

**Table 3:** Surgical procedures and approaches

**Surgical procedure**	Control group n (%)(N = 105)*	Sealant group n (%)(N = 111)*
Parenchymal resections	101 (96.2)	108 (97.3)
Lobectomies	76 (72.4)	74 (66.7)
RUL	29 (27.6)	21 (18.9)
LUL	10 (9.5)	8 (7.2)
ML	5 (4.8)	2 (1.8)
RLL	14 (13.3)	21 (18.9)
LLL	15 (14.3)	20 (18.0)
LB	2 (1.9)	2 (1.8)
UB	1 (1.0)	0 (0.0)
Wedge resections	25 (23.8)	34 (30.6)
Decortications	4 (3.8)	3 (2.7)

*Total exceeds number of patients, as both surgical procedures and approaches are reported.

*RUL* right upper lobectomy, *LUL* left upper lobectomy, *ML* middle lobectomy, *RLL* right lower lobectomy, *LLL* left lower lobectomy, *LB* lower bilobectomy, *UB* upper bilobectomy.

### Efficacy

The incidence of postoperative air leaks, defined as mean transpleural air flow ≥20 ml/min in 24 h with instantaneous air flow peaks ≥200 ml/min, as detected by a digital chest drain system, was significantly lower in the sealant group than the control group (sealant group: 12/111 patients, 10.8%; control group: 19/105 patients, 18.1%; p = 0.0002; Table 4).

**Table 4:** Postoperative outcomes

**Variable**	**Control group (N = 105)**	**Sealant group (N = 111)**	**p-value**
Mean air flow ≥20 ml/min (n,%)	19 (18.1)	12 (10.8)	0.0002
Prolonged air leak (≥5 days) (n,%)	12 (11.4)	3 (2.7)	0.0013*
Mean length of hospital stay (days)	8.4	4.3	0.0001

*Fisher’s exact test.

Prolonged air leaks, defined as a leakage lasting beyond the 5^th^ postoperative day, were recorded in 2.7% (3/111) of patients in the sealant group and in 11.4% (12/105) of patients in the control group. The lower incidence of prolonged air leaks in the sealant group compared with the control group was statistically significant (p = 0.0013; Table 4).

The mean length of hospital stay was significantly shorter in the sealant group than the control group (4 *versus* 8 days; p < 0.0001) (Table 4). The mean number of chest X-rays performed during the hospital stay was three per patient.

## Discussion

Alveolar air leak is generally considered to be the most important complication following lung resection and is the leading cause of postoperative pulmonary morbidity, prolonged length of hospital stay and increased hospital costs.

Intraoperative air leaks following pulmonary resections are reported in 48–70% of cases [15].

There are several studies that have observed that various surgical sealants are safe and effective treatments for intraoperative air leaks following lung resection [16-21]. Three prospective, randomized studies have recently investigated the role of CoSeal® in preventing, or reducing the incidence of alveolar air leaks after lung resection [16,21,22]. Similarly to our study, the results of two of these studies observed that CoSeal® can reduce the incidence and duration of air leaks [16,21], whilst in contrast, the third study did not observe any benefit conferred by the use of CoSeal® [22]. In the first study by Venuta *et al.*[21], 50 patients undergoing standard pulmonary lobectomy with incomplete or absent fissures were intraoperatively randomized into two groups: group I received CoSeal®, applied over the newly-designed fissures; group II received no sealants. The groups differed with respect to duration of drainage, hospital stay, and presence of air leakage during the first five days. A PAL (>7 days) was present in 8% and 20% of patients in group I and group II, respectively. The authors concluded that the use of CoSeal® in selected cases may help to reduce the incidence and duration of air leaks [21].

The second study by D’Andrilli *et al.*[16] evaluated the effectiveness and safety of CoSeal® in reducing air leaks in patients undergoing lung resection with reinforcement of the stapled line by bovine pericardial strips in case of incomplete fissure. In total, 203 patients undergoing anatomic or atypical lung resection were enrolled. Patients showing moderate or severe air leaks were intraoperatively randomized to either the standard care group (suture-stapling) or the CoSeal® group (suture-stapling plus CoSeal®). The intraoperative air leak cessation rate was significantly higher in the CoSeal® group compared with the standard care group. In addition, the CoSeal® group showed a significantly lower rate of air leaks after 24 and 48 h [16]. The application of CoSeal® sealant proved effective in reducing air leaks and in shortening the duration of PAL.

The third study by Tan *et al.*[22] evaluated the effectiveness of CoSeal® in reducing the duration of air leaks in patients undergoing lung resection. Patients who experienced an intraoperative air leak during the underwater air-tightness test were randomized to either the CoSeal® group or standard care group. At 24 h, there was no difference in air leak between the groups and fewer patients in the control group were leaking at 48 h postoperatively [22].

Possible reasons for the conflicting results of this study [22] with those of D’Andrilli *et al.*[16], Venuta *et al.*[21] and our study, could be due to the inclusion of approximately 40% of the patient poulation in the Tan *et al.* study with mild (Grade 1) intraoperative air leaks, which usually have a rapid, spontaneous resolution, and differences in the dose of product applied in each patient [23]. Additionally, in contrast to our study, postoperative air leaks in these studies [16,21,22] were detected by an analogical chest drainage system, so air leaks could not be accurately measured. The detection of air leaks has recently been improved by the addition of digital units to chest drains. The prototype digital chest drain is DigiVent™, which consists of a collection chamber, equipped with a unidirectional dry valve, digital displays, and software that can assess and record instantaneous and cumulative air flows and pleural pressures. In our experience, the use of digital chest drain systems in patients undergoing parenchymal resection, or other procedures in which sealants have been applied, has yielded exciting results.

In our study, the incidence of air leaks detected by DigiVent™ was significantly lower in patients treated with CoSeal® compared with controls (10.8% *versus* 18.1%, respectively). Moreover, there was a significantly higher incidence of prolonged air leaks in the control group compared with the CoSeal® group (11.4% *versus* 2.7%, respectively). The mean hospital stay was shorter amongst patients treated with CoSeal® compared with controls, which may translate into associated cost savings. In the present study, CoSeal® demonstrated superior air sealing efficacy compared with standard care in patients with good pulmonary function, showing a significantly reduced proportion of patients with air leaks and lower mean air leak volume.

CoSeal® may be expected to be even more effective in high-risk patients who may be predisposed to fragile, poor quality lung parenchyma (e.g., patients with chronic obstructive pulmonary disease ([COPD], inflammation or apical fibrosis). However, further stratification of patients is required in order to evaluate the advantages of continuous digital recording of air leaks following sealant application in high-risk cohorts (e.g. COPD) and patients undergoing upper lobe resections. These studies could also include investigations relating to the extent of parenchymal resection (in terms of suture line length) and the characteristics of staplers. Such studies would provide interesting information concerning the prevention, early recognition and treatment of air leaks in a larger number of patients.

## Conclusions

The results of this study suggest that CoSeal® is an effective method of reducing postoperative alveolar air leaks, continuously monitored using the DigiVent™ system, in patients undergoing elective lung resection. In our opinion, the use of CoSeal® could have a favorable impact on patient compliance, as well as reducing the length of hospital stays and associated costs.

## Abbreviations

COPD: Chronic obstructive pulmonary disease; CT: Computed tomography; FEV_**1**_: Forced expiratory volume in one second; LB: Lower bilobectomy; LLL: Left lower lobectomy; LUL: Left upper lobectomy; ML: Middle lobectomy; paCO_2_: Partial pressure of carbon dioxide; paO_2_: Partial pressure of oxygen; PAL: Prolonged air leaks; PEG: Polyethylene glycol; ppoFEV_**1**_: Predicted postoperative FEV_**1**_; RLL: Right lower lobectomy; RUL: Right upper lobectomy; UB: Upper bilobectomy.

## Competing interests

No sources of funding were used to support the study. The authors had full control of the design of the study, methods used, outcome parameters and results, analysis of data and production of the written report. All authors declare that they have no competing interests.

## Authors’ contributions

CL participated in the design of the study and its coordination and helped to draft the manuscript. GG participated in the design of the study and helped to draft the manuscript. RM participated in the design of the study, participated in the sequence alignment and helped to draft the manuscript. ADM participated in the design of the study, participated in the sequence alignment and helped to draft the manuscript. MG performed the statistical analysis. All authors read and approved the final manuscript.
